# Reasoning the Causality of City Sprawl, Traffic Congestion, and Green Land Disappearance in Taiwan Using the CLD Model

**DOI:** 10.3390/ijerph111111464

**Published:** 2014-11-06

**Authors:** Mei-Chih Chen, Kaowen Chang

**Affiliations:** Department of Landscape Architecture, National Chiayi University, No. 300 Syuefu Rd., Chiayi City 60004, Taiwan; E-Mail: kaowen@mail.ncyu.edu.tw

**Keywords:** reasoning, causality, city sprawl, land use management, causal loops diagram (CLD)

## Abstract

Many city governments choose to supply more developable land and transportation infrastructure with the hope of attracting people and businesses to their cities. However, like those in Taiwan, major cities worldwide suffer from traffic congestion. This study applies the system thinking logic of the causal loops diagram (CLD) model in the System Dynamics (SD) approach to analyze the issue of traffic congestion and other issues related to roads and land development in Taiwan’s cities. Comparing the characteristics of development trends with yearbook data for 2002 to 2013 for all of Taiwan’s cities, this study explores the developing phenomenon of unlimited city sprawl and identifies the cause and effect relationships in the characteristics of development trends in traffic congestion, high-density population aggregation in cities, land development, and green land disappearance resulting from city sprawl. This study provides conclusions for Taiwan’s cities’ sustainability and development (S&D). When developing S&D policies, during decision making processes concerning city planning and land use management, governments should think with a holistic view of carrying capacity with the assistance of system thinking to clarify the prejudices in favor of the unlimited developing phenomena resulting from city sprawl.

## 1. Introduction

Many city governments in Taiwan believe that developing rural areas and non-classified areas (*i.e*., green land) and improving the city transportation network can improve a city’s economy. For the last 60 years, the positive effects of economic growth have made many of Taiwan’s cities prosperous. However, the negative effects, such as city sprawl, high-density population aggregation in cities, traffic congestion, and the disappearance of green land, persist in Taiwan’s major cities [1]. These issues affect many major cities worldwide, and most city governments and people in cities believe that economic growth is essential for city growth. In terms of the positive and negative effects of economic development in cities [2,3], city sprawl should be a focus of city governments and those authorities related to environmental planning and management. Studies have highlighted the urgent need to watch out for the disappearance of green land from city sprawl, because the carrying capacity of the ecological function supplied by green land which benefits the healthy lives of people [4,5,6,7,8].

People generally believe that providing more developable land and transportation infrastructure will benefit the economic activities of cities and solve the development and traffic congestion problems [1,9,10]. Trends for all cities in Taiwan over the last 60 years reveal that the unlimited developable land means the loss of city growth, which has both positive and negative effects, even though most Taiwan cities aim for sustainability and development (S&D). These un-intuitional effects of city development means the elements in cities have causality, which influences the behaviors and system structures of cities [3,9,11,12,13,14].

This study compares the characteristics of development trends with yearbook data for 2002 to 2013 for all of Taiwan’s cities, applies the causal loops diagram (CLD) (also called the feedback loops diagram) of the System Dynamics (SD) approach to reason the causality in land development, high-density population aggregation in cities, traffic congestion, and green land disappearance due to development. The system thinking logic in the SD approach helps to identify characteristics of the dynamic interactions among elements and system structures related to city development [15,16], and explore the demand of the holistic view of carrying capacity on policy decision of land use to assist Taiwan’s cities achieve sustainability and development (S&D).

The rest of this paper is organized as follows: using the SD approach, Section 2 introduces the conditions of development in Taiwan’s cities from 2002 to 2013. Section 3 presents the reasoning of CLD models to analyze the causality on issue of traffic congestion and those issues related to land development in Taiwan’s cities. Section 4 then applies two CLD models to the issues of city traffic congestion and those related to land development in Taiwan’s cities: general development based on view of economic growth; and S&D development with a view of carrying capacity. This section discusses reasoning results by the CLD models for city development trends. Finally, some conclusions regarding city development in relation to the carrying capacity perspective supported by the SD approach are given. These conclusions address city development and land development problems in the context of the S&D city.

## 2. Development of Taiwan’s Cities from 2002 to 2013

### 2.1. Land Development, Green Land, and Population

Taiwan is a long mountainous island with numerous mountain ranges running north to south. The mountainous areas occupy 73% of Taiwan’s landmass and the plains account for 27% of land [1,17]. Taiwan’s major cities are located on the plains, in basins, and on tablelands. Based on yearbook data for cities in Taiwan from 2002 to 2013, major cities have high population densities [1,17]; especially Taipei City and New Taipei City which are leading ahead (Table 1). In 2013, the ratio of the population living in Taiwan’s cities increased by approximately 80% and Taipei City’s relative population ratio is 100%, meaning that the number of person/km^2^ in cities increased by 3940 persons (population density in Taipei is 9880 persons/km^2^; 7th highest density worldwide). That is, 1,124,000 persons (roughly 6.4% of the city population in Taiwan in 2002) were added to cities or migrated to cities from rural areas from 2002 to 2013 (Table 1).

**Table 1:** City development of Taiwan, 2002 to 2013.

Item	Year-End 2002	Year-End 2013	Increase	Percentage
Population of City Area	17,661,000	18,785,000	1,124,000	6.4
Ratio of City Population	78	80	2	2.6
Population Density of City Area	3760	3940	180	4.8
City Planning Area	4680	4760	80	1.7
Rural Area	22,600	29,100	6,500	28.8
Ratio of Rural Population	21	19	−2	−9.5
Non-Classified Area ^1^	8880	2230	−6650	−74.9
Total Land in Taiwan	-	36,192	-	-
Planning Area in New Taipei City	1,200	1240	40	3.3
Planning Area in Taipei City	271	271	0	0
Population in New Taipei City	3,371,000	3,744,000	373,000	11.1
Population in Taipei City	2,641,000	2,686,000	45,000	1.7
Population Density in New Taipei City	2780	3000	220	7.9
Population Density in Taipei City	9720	9880	160	1.6

Source: This table was produced by this study and arranged from [1,17]; ^1^ The “Non- Classified Area” is defined to lands not yet classified, meaning “Green Land” in this study. (Unit: person, km^2^, persons/km^2^, %).

Additionally, in response to the demands of residents living in high-density areas, and needed transportation infrastructure for economic growth, city governments choose short-term policies like developing rural areas and non-classified areas, which are generally called “Green Land” in this study. These policies satisfy the demand of city development by easing restrictions on rural and non-classified lands, which immediately offering large areas for roads, public facilities, and living space [9,18]. Accordingly, 78.33 km^2^ of non-classified and rural areas have been identified as the city’s planning area. Moreover, 6650 km^2^ of non-classified areas (74.9% of the non-classified areas in Taiwan in 2002) have been transformed into city planning areas and rural areas during 2002 to 2013. These short-sighted policies bring unlimited development and city sprawl, typical of most Taiwan cities (Table 1).

### 2.2. Traffic Congestion, Road Construction, and Motor Vehicle Users

In terms of traffic congestion in cities, the high density of motor vehicles in cities in Taiwan, which is generally called “Traffic Congestion” in more recent CLD models, is usually controlled by controlling the number of motor vehicles and volume of road areas. Additionally, the number of Motor Vehicles usually increases as the number of users increases, which is usually closely related to Population of City Area and City Households (Table 1 and Table 2). Particularly, the number of Motor Vehicles is usually counted by number of City Households. The yearbook data reveals that the average number of motor vehicles per household is 2.6 cars during 2002 to 2013 [17].

**Table 2:** Road construction and motor vehicles development in Taiwan’s cities from 2002 to 2013.

Item	Year-End 2002	Year-End 2013	Increase	Percentage
Motor Vehicles	17,906,000	21,562,000	3,655,000	20.4
City Households	6,925,000	8,286,000	1,361,000	19.7
Motor Vehicles per Household	2.58	2.60	0.01	0.8
Motor Vehicle Density ^1^	38	45	7	18.4
Road Areas	40,200	50,900	10,700	26.7
Road Length	37,000	42,500	5500	14.9

Source: This table was produced by this study and arranged from [1,17]; ^1^ Motor Vehicle Density equals the number of motor vehicles divided by city planning area in Taiwan. (Unit: hectare, household, car, cars/hectare, km, %).

Moreover, in 2013, the percentage increase in number of city households in Taiwan was 19.7% (8,286,000 households) and the percentage increase in number of motor vehicles was 20.4% (21,562,000 cars), confirming the relationship between Motor Vehicles and City Households (Table 2). The increase in population and number of households increased the density of motor vehicle in Taiwan’s cities from 38 to 45 cars per hectare (18.4% in 2002) (Table 1 and Table 2).

## 3. Traffic Congestion Development Reasoning, CLD Models

The yearbook data (Table 1 and Table 2) show that most negative effects like city sprawl (“City Planning Area” increasing), high-density population aggregation in cities (“Population Density of City Area” increasing), traffic congestion (“Motor Vehicle Density” increasing), and the disappearance of green land (Non-Classified Area decreasing) of city development have increased in severity. In particular, developing more green land into city planning areas for roads for motor vehicle users, did not control city traffic congestion during 2002 to 2013. The cause and effect of city development policies did not follow the rule that one policy solves one issue. Furthermore, traffic congestion is a major issue for cities worldwide. This study takes Taiwan’s cities as reasoning cases and applies CLD models of the SD approach and city traffic congestion as the main topic to explore the un-intuitional positive and negative effects of city sprawl. Furthermore, this study also reasons the causality in traffic congestion and other issues relevant to roads and land development, high-density population aggregation in cities, and green land disappearance from cities in Taiwan. The CLD models are discussed as follows:

### 3.1. The General Logical CLD Model for Cause and Effect of Traffic Congestion

**Figure 1:**
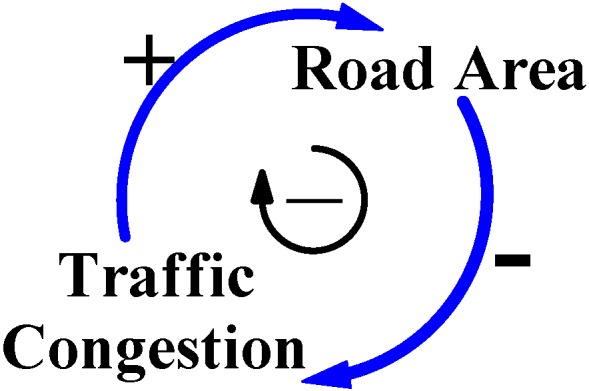
This CLD model shows the general logic when reasoning city traffic congestion.

Source: This CLD model was produced and constructed by this study and referred to [9].

In Taiwan, city governments are always concerned with economic benefits during any decision process concerning city development. Their basic principles of policy assessment are simple, conducted easily, and cost effective; those policies quickly decrease the symptomatic degree of negative effects of city development to benefit the city economy. Consequently, to reduce “Traffic Congestion”, most city governments believe that increasing the “Road Area” may fit with their policy principles and address quickly the symptomatic degree of traffic congestion [9]. This thinking logic is illustrated by the left positive arrow loop and the right negative arrow loop in Figure 1; a larger volume of road area and city traffic congestion would be eliminated. Figure 1 is a CLD model that shows feedback relationships between the two causal variables, Road Area and Traffic Congestion.

**Figure 2:**
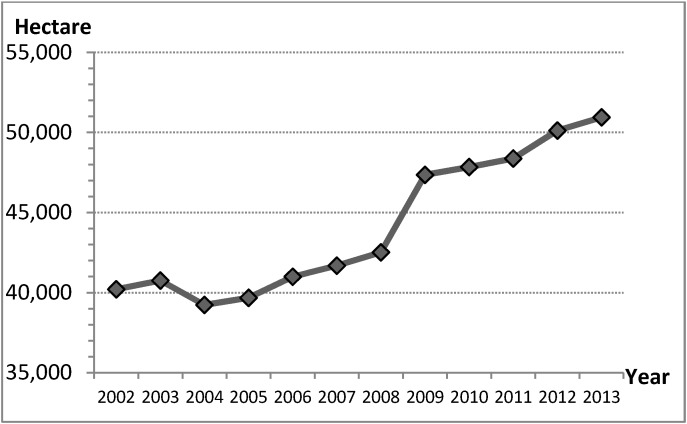
Trends for road areas construction, Taiwan’s cities, 2002 to 2013.

Source: This figure was produced by this study and arranged from [1].

The policy of increasing the supply of road areas by city governments is clearly reflected in road areas, road length and trends for road areas construction (Table 2 and Figure 2). These show statistical data for road construction development in Taiwan’s cities, and also reveal that road construction was *via* the decision process for city development from 2002 to 2013 [1,17]. The percentage increase in road areas was 26.7% and road length was 14.9% in 2013, meaning that the road areas increased by 50,900 hectares and the road length increased by 42,500 kilometers in Taiwan’s cities (Table 2). These data and developing trends for road areas construction (Table 2 and Figure 2) confirm that the policy increases the supply of road areas to address traffic congestion. This logic for city traffic congestion and the statistic data (Table 2) are illustrated by CLD model in Figure 1.

**Figure 3:**
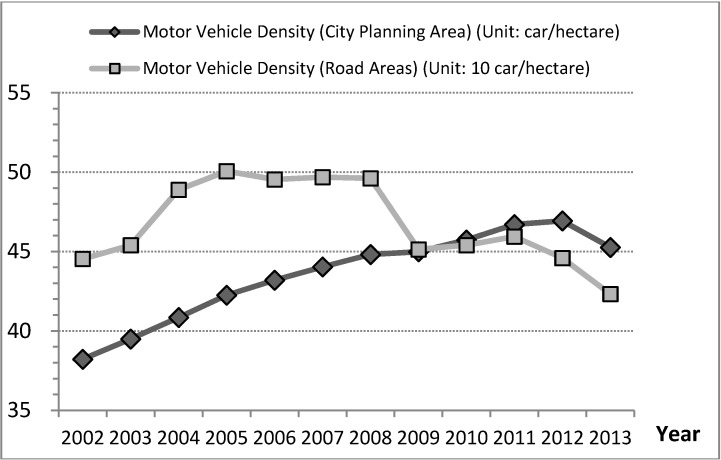
Trends for motor vehicle density, Taiwan’s cities, 2002 to 2013.

Source: This figure was produced by this study and arranged from [1,17].

However, this reasoning does not conform to the development trends for motor vehicle density. If the denominator of motor vehicle density was road areas, traffic congestion would have been alleviated in 2008 and the percentage decrease in the density of motor vehicles was −0.5% (−2.2 cars/hectare); however, if the denominator of motor vehicle density was city planning area, the development trends of motor vehicle density continued increasing and the percentage increase in the density of motor vehicles was 18.4% (7 cars/hectare) in cities during 2002 to 2013 (Figure 3 and Table 3). Thus, traffic congestion was not alleviated by the policy increasing the road areas from 2002 to 2013. This discovery is differing from decision logic of treatment by city governments that supplying more road areas decreases the density of motor vehicles and alleviates traffic congestion, which benefit city economic activities.

**Table 3:** Density of motor vehicles, household, and road construction development in Taiwan’s cities, 2002 to 2013.

Item	Year-End 2002	Year-End 2013	Increase	Percentage
Motor Vehicles	17,906,000	21,562,000	3,655,000	20.4
Motor Vehicles in New Taipei City	2,643,000	3,233,000	590,000	22.3
Motor Vehicles in Taipei City	1,649,000	1,802,000	153,000	9.3
City Households	6,925,000	8,286,000	1,361,000	19.7
Households in New Taipei City	1,190,000	1,477,000	287,000	24.1
Households in Taipei City	906,900	1,026,700	119,800	13.2
Motor Vehicle Density 1 ^1^	38	45	7	18.4
Motor Vehicle Density 2 ^1^	445.3	423.2	−2.2	−0.5
City Planning Area	4680	4760	80	1.7
Road Areas ^1^	40,200	50,900	10,700	26.7
Road Length	37,000	42,500	5500	14.9

Source: This table was produced from this study and arranged from [1,17]; ^1^ “Motor Vehicle Density 1” is the number of motor vehicles divided by the city planning area, and “Motor Vehicle Density 2” is the number of motor vehicles divided by road areas in cities in Taiwan. (Unit: car, cars/hectare, hectare, km, %).

### 3.2. The Main CLD Model for Cause and Effect of Traffic Congestion

Furthermore, the percentage of motor vehicles increased with the number of users of motor vehicles, which is usually rooted in the population of the city area and city households (Table 1, Table 2 and Table 3). From 2002 to 2013, data for Taipei City and New Taipei City also reveal that users of motor vehicles include the active population that comes from neighboring cities. For instance, New Taipei City neighbors Taipei City, and the percentage increase in the population of New Taipei City was 11.1%, higher than that of Taipei City at 1.7% from 2002 to 2013 (Table 1). Further, the percentage increase to households was 24.1% in New Taipei City and 13.2% in Taipei City, and the percentage increase to motor vehicles was 22.3% in New Taipei City and 9.3% in Taipei City (Table 3). These two cities have a very close relationship.

**Figure 4:**
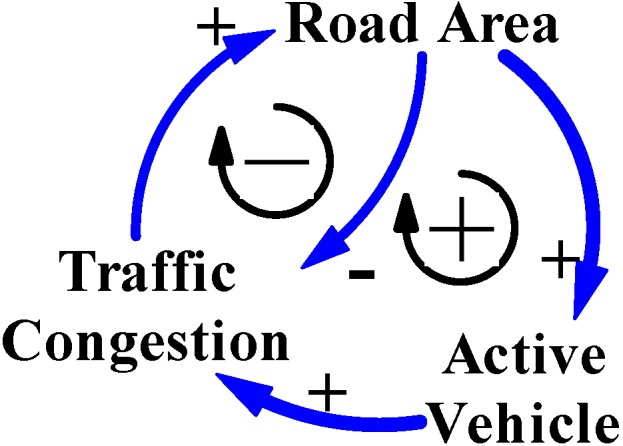
The CLD model reasons the main cause-and-effect variables for city traffic congestion in Taiwan’s cities.

Source: This CLD model was produced and constructed by this study and referred to [9,19].

Yearbook data for Taipei City and New Taipei City confirm the mentioned phenomenon that the number of motor vehicles is influenced by the active population, including users of motor vehicles in the city and those from neighbor cities. Therefore, motor vehicle is another key variable that influences symptomatic traffic congestion, which is generally called “Active Vehicle” in the CLD models in this study. Figure 4 shows the CLD model illustrating the main structure of cause-and-effect variables for city traffic congestion in Taiwan.

## 4. Two Treatments of Traffic Congestion Reasoning, CLD Models

Learning from the economic growth of American cities, many people believe that the main task of land use management by city governments is to divide the city in rarely used green land into areas with great economic benefits for industry, commerce, public transportation, residences, leisure, and public services. This provides a satisfying, sufficient, and safe environment for people [18,20,21]. However, the greatest benefit is improving the city economy. Likewise, when city governments in Taiwan make policy decisions for land use and city planning, they prioritize economic growth. This logic has become the standard goal for city development.

However, land and environmental resources on developed plain areas, on which most cities are located, have different carrying capacities. City planning areas need green land with good “Land Capacity” to recover, regulate, and preserve eco-functions such as the circulations of air, water, and various natural resources [16,20,21]. In other words, land use and development policies should be sustainable in terms of various carrying capacities rather that concentrate only on economic growth [16,21,22]. These differing viewpoints influence trends for city traffic congestion and development. The following two treatments of traffic congestion reasoning by CLD models are discussed as follows.

### 4.1. Economic Growth

**Figure 5:**
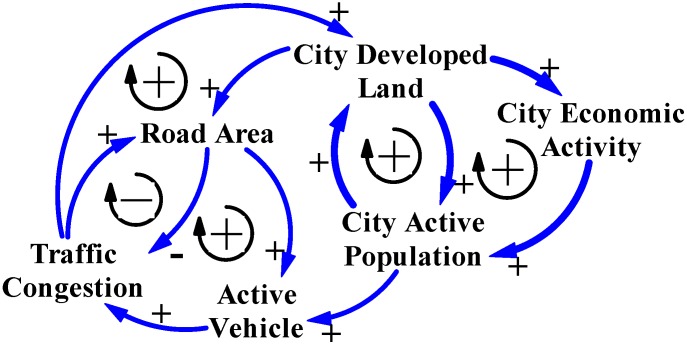
The CLD model reasons three key variables: City Active Population, City Developed Land, and City Economic Activity. All variables directly influence the dynamic relationships between city development and traffic congestion in Taiwan’s cities.

Source: This CLD model was produced and constructed by this study.

Generally, as the demand for “Road Area” increases as a treatment for traffic congestion, city governments would satisfy by offering large amounts of “City Developed Land” for roads. The logic of governments in their land use policies and their positive effects on city traffic congestion not only satisfy the demand for road areas by city active population and industries, but may also promote “City Economic Activity”. These city governments’ thinking logic are indicated by the right upward positive arrow loops on the CLD model (Figure 5), which reasons the cause-and-effect feedback relationships among three causal variables: Road Area, City Developed Land, and City Economic Activity.

When city governments have sufficient road areas to eliminate traffic congestion, positive experiences will increase the number of vehicle users (the “City Active Population” in the CLD models) and satisfy demand for increased city economic activities. This behavior of the city active population is indicated by the right downward positive arrows in the CLD model in Figure 5, which reasons the cause-and-effect feedback relationships among three causal variables: Active Vehicle, City Active Population, and City Economic Activity.

Compared to rural areas, cities typically have economic advantage and often attract people from rural areas [18]. This is reflected by the ratio of the population living in Taiwan’s cities increased by 80% in 2013 (Table 1) [1,17]. Therefore, governments always increase their supply of city developed land by using “City Planning Area” to reduce the density of the city active population. However, the cause-and-effect relationships among variables: City Planning Area and “Population Density of City Planning Area” are delayed.

**Figure 6:**
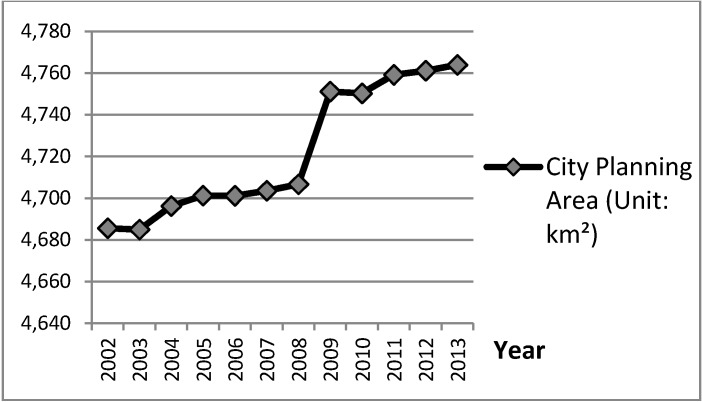
Trend for “City Planning Area” in Taiwan’s cities, which directly reveals the cause-and-effect relationships between variables of City Developed Land and City Active Population, 2002 to 2013.

Source: This figure was produced by this study and arranged from [1,17].

**Figure 7:**
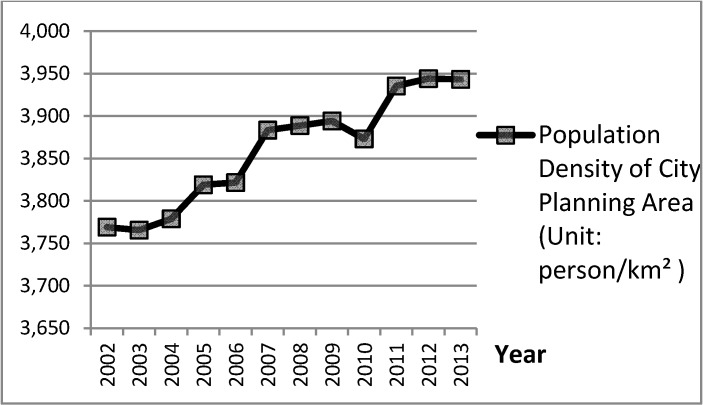
Trend for “Population Density of City Planning Area” in Taiwan’s cities, which directly reveals the cause-and-effect between variables of City Developed Land and City Active Population, 2002 to 2013.

Source: This figure was produced by this study and arranged from [1,17].

Figure 6 and Figure 7 show statistical graphs of city planning area plotted against city population density from yearbook data for Taiwan’s cities from 2002 to 2013. Compared to their development trends, when the developing trends of variable City Planning Area were upward in 2003 to 2005, 2008 to 2009, and 2010 to 2011, the developing trends of variable Population Density of City Planning Area would slow after the periods 2005 to 2006, 2007 to 2009, and 2012 to 2013. Specifically, the great upward developing trend of variable City Planning Area during 2008 to 2009 resulted in the clear downward developing trend of variable City Population Density in 2009 to 2010. These delayed cause-and-effect phenomena between variables of City Developed Land and City Active Population, which are reasoned by the CLD model in Figure 5.

**Figure 8:**
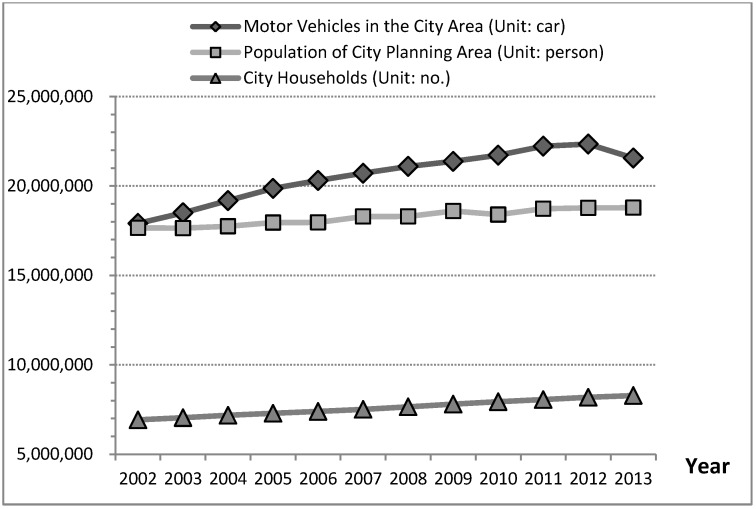
Trends for motor vehicles in the city area, population of city planning area, and city households, Taiwan’s cities, 2002 to 2013.

Source: This figure was produced by this study and arranged from [1,17].

Furthermore, the increased volume of road areas would also satisfy demand from vehicle users. These users are generally called the City Active Population in CLD models in this study. Accordingly, a large active population (employment, study, tourism, and other) is often attracted to cities, causing the number of active vehicles to increase for most of the population and households in a city area that use vehicles. This phenomenon is defined as the city active population increasing trend increases the number of motor vehicle in cities. The development trend of motor vehicles in the city area is closely related to development trends of population of city planning area and city households from 2002 to 2013 (Figure 8), which fits with data and reasons (Table 2 and Figure 5). Therefore, variables Motor Vehicles and Population of City Planning Area are called “Active Vehicle” and “City Active Population” in the CLD models in this study.

**Figure 9:**
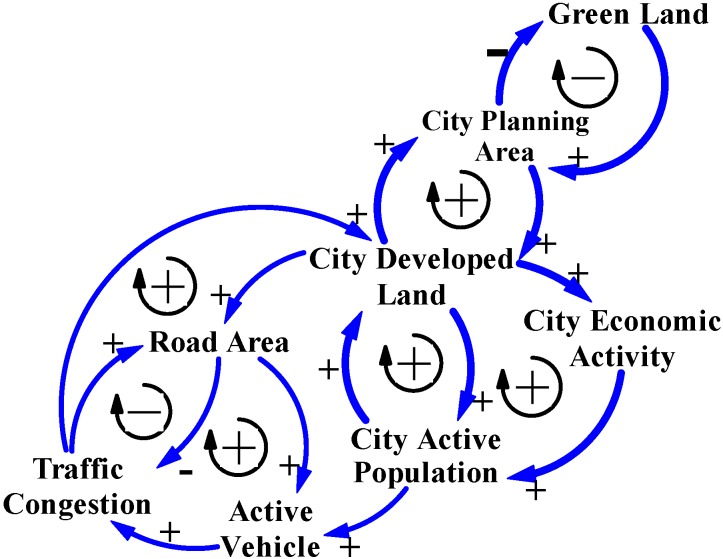
The positive and negative feedback loops of the CLD model in the context of economic growth for reasoning the relationships among land development, green land disappearance, and traffic congestion.

Source: This CLD model was produced and constructed by this study.

On the basis of these cause-and-effect relations within key variables for city traffic congestion, when the volume of developed land was insufficient for development of the road areas and other land uses, city governments continually develop green land into developed land to satisfy the increased demand for road areas and other land uses in cities [15,16,18,20,23]. These causalities treat city development and traffic congestion with the context of economic growth have dominated for many decades, often resulting city sprawl in Taiwan [18]. This phenomenon has reasoned positive and negative effects (Figure 9) and fit with yearbook data for 2002 to 2013 (Table 1), indicating that there are 80 km^2^ of green land have been developed into the city planning area, and also 6650 km^2^ of green land (74.9% of green land were originally the non-classified areas in Taiwan in 2002) have been developed into rural area and parts for city planning area from 2002 to 2013 in Taiwan.

### 4.2. Carrying Capacity

**Figure 10:**
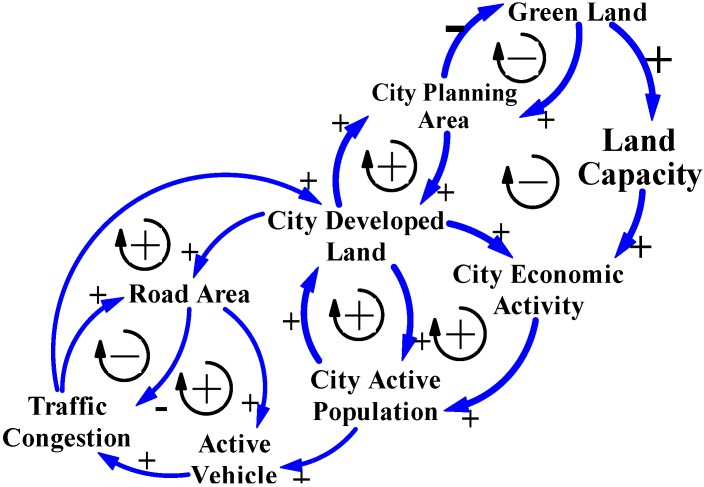
The causality of positive and negative feedback loops in the CLD model indicate that governments face development and traffic congestion problems when they consider eco-city principles.

Source: This CLD model was produced and constructed by this study.

In terms of a holistic ecosystem, policies that treat traffic congestion and city development by increasing the amount of available land for benefit the economic growth usually look to green land. Ecological carrying capacity (Land Capacity in CLD models) influences the health of eco-functions and conservation of natural resources in an ecosystem. Therefore, green land should be preserved and protected by city governments (Figure 10) [2,16,21,24].

Furthermore, the land capacity of green land in cities has accumulative power to supply sustainable natural resources, fresh air and sunshine for leisure activities and exercise, citizens’ farm, and other eco-functions that maintain steady city economic activity [16]. These benefits of green land are very important and usually considered critical indicators of an S&D city. Therefore, green land, such as parks, greenbelts, plazas and other public spaces, have been constructed by city governments in recent decades. There were 5600 hectares of green land in five metropolises, occupying nearly 70% of the total green land in Taiwan in 2013. Compared to the 4130 hectares of green land in 2002, the increase by 2013 was nearly 94% at 3870 hectares (Table 4).

**Table 4:** Green Land constructed by five metropolises in Taiwan in 2002 to 2013.

Item	New Taipei City	Taipei City	Taichung City	Tainan City	Kaohsiung City	Sum of Five Metropolises	Total Number of Cities in Taiwan
Green Land ^1^ in 2002	440	640	360	280	850	2590	4130
	(10.7) ^2^	(15.5)	(8.7)	(6.8)	(20.6)	(62.7)	(100.0)
Green Land ^1^ in 2013	800	710	980	800	2290	5600	8000
	(10.0) ^2^	(8.9)	(12.3)	(10.0)	(28.6)	(70.0)	(100.0)
Increased Area	360	70	620	520	1440	3010	3870
	(81.8) ^3^	(10.9)	(172.2)	(185.7)	(169.4)	(116.2)	(93.7)

Source: This table was produced by this study and arranged from [1,17]; ^1^ Green land includes parks, greenbelts, plazas, playgrounds, and stadiums; ^2^ The number inside the parentheses is the ratio of green land in a city to that in all cities in Taiwan. ^3^ The number inside the parentheses is the increase ratio of green land in a city from 2002 to 2013. (Unit: hectare, %).

**Figure 11:**
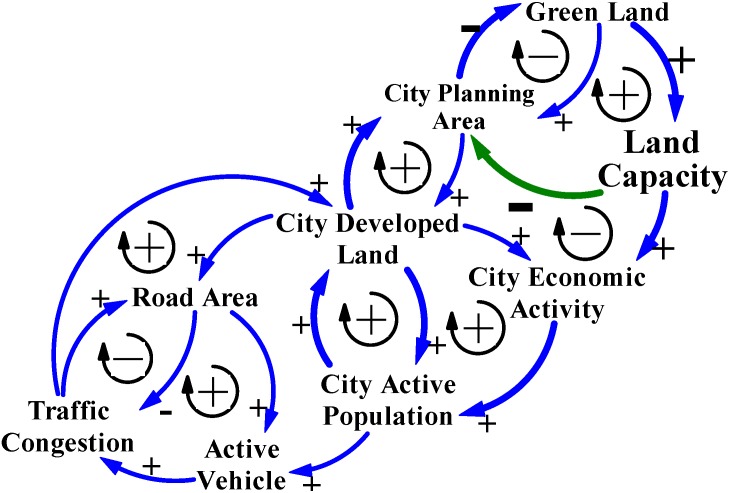
The conceptual S&D CLD model for reasoning city development and traffic congestion policies based on carrying capacity.

Source: This CLD model was produced and constructed by this study.

City governments in Taiwan rarely preserve the original green land for their eco-city status, and the green land trend means that the ecological carrying capacity (Land Capacity in CLD models in Figure 10 and Figure 11) of green land in cities does influence the health of eco-functions of city ecosystems or the people who, say, live, work, study, and visit cities. Therefore, city governments often construct the artificial green land to offset the vast amount of green land that was developed as city developed land (Table 4). The positive and negative feedback loops in the CLD models indicate that many governments face city development dilemmas when they consider eco-city principles, reasoning that land use policy in Taiwan has causal relations with traffic congestion, green land disappearance, and unlimited demand for city developed land.

There are other interesting factors related to city sprawl (*i.e.*, city economic growth) in Taiwan. During the developing processes for 22 cities in Taiwan (Table 5), the present numbers for 20 cities’ population and 18 cities’ population density were both leaving from the anticipated city plans from 2002 to 2013. In total, 15 cities still increase their city planning areas and the present population density of the major cities were leaving from the anticipated city plans (Table 5) [1,17].

**Table 5:** The factors related to city sprawl for Taiwan cities approaching or leaving the anticipated city plans on population, population density, and city planning area; comparing the annual surplus revenue and expenditures from 2002 to 2013.

Item	City	County ^1^	Total ^1^
City Planning Area Decreased	1	5	6
City Planning Area Increased	6	9	15
City Planning Area Unchanged	1	0	1
Population Leaving from Anticipated City Plans	8	12	20
Population Approaching to Anticipated City Plans	0	2	2
Population Density Leaving from Anticipated City Plans	6	12	18
Population Density Approaching to Anticipated City Plans	2	2	4
Negative Annual Surplus Revenue and Expenditures	5	11	16
Positive Annual Surplus Revenue and Expenditures	3	3	6

Source: This table was produced by this study and arranged from [1,17]; ^1^ The scale of development in counties was considered to cities’ scale in Taiwan; therefore, the total number of cities is 22. (Unit: number).

Moreover, since city governments believe that city sprawl means economic growth, the positive trends of annual surplus revenue and expenditures that based on yearbook data for cities in Taiwan from 2002 to 2013, which numbers of cities should correspond with cities increased their planning areas (Table 5). However, only six cities had some positive effects on the annual surplus revenue and expenditures among 15 cities that planning areas increased [1,17]. This economic growth by city development in Taiwan (Figure 10) is contrary to the beliefs of city governments increasing the amount of developed land benefit economic activities. Unlimited growth challenges the carrying capacity of cities and the manpower of city governments to administer affairs of S&D city development and land use management of Taiwan, both of which are needed to balance supply and demand issues and keep city development processes stable [16]. Accordingly, city governments in mountainous Taiwan should not only rely on extending the city planning territory as the only path for city economic growth and development. In other words, governments should admit that land and environmental resources are not unlimited, and that the carrying capacity and environmental resources of green land in mountainous ecosystems should be of concern when making land use policy [16]. Figure 11 shows the conceptual S&D CLD model for reasoning city development and traffic congestion policies based on carrying capacity.

On the strength of the conceptual S&D CLD model (Figure 11), city governments should have power to stop green land from becoming a city planning area and later city developed land. Cities in Taiwan could avoid city sprawl. Moreover, based on the limited amount of plain areas in Taiwan, as cities aim to become S&D cities by preserving and restraining development of green land, these cities would have increased opportunities to find policies and methods based on carrying capacity (Figure 11).

Furthermore, policies that preserve green land could help when managing contingencies by adjusting the intensity and density of land use to control the city active population [16]. Therefore, the number of active vehicles would be controlled and traffic congestion in cities will be eliminated (Figure 11). Furthermore, land use policies based on carrying capacity could also cultivate demands for a healthy environment. These long-term policies are necessary for cities that plan to become S&D cities.

## 5. Conclusions

All cities in Taiwan are located on limited land in this mountainous country. If city development policies are not concerned with carrying capacity and only focus on economic growth, traffic congestion and other city problems will easily develop, including unlimited city sprawl. This unlimited growth challenges cities that are based on a natural ecosystem and the manpower of city governments to administer the affairs of S&D city development and land use management of Taiwan, which must be balanced to remain stable. However, traffic congestion, high-density population aggregation, significant destruction, pollution, green land disappearance, and environmental collapse have existed for at least a few decades, and there are sufficient proofs that current development policies were developed by governments without concern for carrying capacity. These are significant challenges as Taiwanese cities seek to become S&D cities.

Logic of causality supported by the CLD models of the SD approach reasoned the causal problems of traffic congestion, green land disappearance, and limited developed land for Taiwan’s cities, and can assist decision groups and city governments in considering carrying capacity. The major findings and the scope and further work obtained by this study are as follows.

### 5.1. City Sprawl is Not Synonymous with S&D Economic Growth

The extent of governments’ visions for development determines city development policies. Unlimited development of green land positively affects economic growth, and also generates city sprawl, high population density, green land disappearance, and traffic congestion. The causalities of these effects have influenced elements and structures of cities in Taiwan for many decades. City governments have always paid the most attention to manpower for management and maintenance, and public expenditures for maintaining public facilities. Therefore, governments should admit that green land, natural resources, and manpower are not unlimited, and they should challenge the idea that city sprawl is synonymous with the S&D of economic growth. The limited carrying capacity and mountainous ecosystem of green land in Taiwan’s cities should be concerns for land use policies for city development.

### 5.2. City Sprawl and More Roads Will Not Necessarily Alleviate Traffic Congestion

City governments in Taiwan should challenge the viewpoint that city sprawl and more roads are necessary to alleviate traffic congestion. As the positive and negative feedback loops of the CLD model reasons, traffic congestion, green land disappearance, and limited developable land have interactive influences. Therefore, when a policy is implemented by a city government to treat one of these issues like alleviating traffic congestion, which policy of increasing city developed land to increase the land available for road area results in green land reductions and city active population increases, such that the number of active vehicles increase as the number of users (*i.e.*, City Active Population) increase, which would result in traffic congestion development again. Yearbook data show these causalities. Therefore, city sprawl (additional developable lands) and increasing the number of roads will not necessarily reduce city traffic congestion.

### 5.3. Causality Reasoning and Logical System Thinking of the CLD Models of the SD Approach in Light of Carrying Capacity Helps Alleviate City Sprawl and Traffic Congestion

Major cities in Taiwan have developed green land to increase developed land with the aim of improving economic growth for many decades. City governments have always satisfied the demands of the industry, vehicle users, and residents. However, the developing trends are far from the anticipated for cities’ plans, which are contrary to the beliefs of city governments of Taiwan that supply more developed lands and transportation infrastructure would benefit cities’ economy activities.

For cities that aim to become S&D cities, governments must recognize the fact that land and environmental resources are not unlimited. By using the CLD model of the SD approach to reason causality, the limited carrying capacity and environmental resources of green land and manpower for environmental management and maintenance should be a concern of land use policy (Figure 11). The S&D solutions and policies, such as using quotas for land development to preserve green land and limit developed land, renovating inferior areas, applying urban design regulations and other regulations for land use and environmental management, would help control city sprawl and help cities in Taiwan become S&D cities.

### 5.4. Scope and Further Work

This study applies the system thinking logic of the CLD models in the SD approach to analyze and explore the unlimited developing phenomena from city sprawl to the issues of traffic congestion, land development, high-density population aggregation in cities, and the disappearance of green land in Taiwan’s cities. In terms of reasoning and identifying the cause and effect relationships in the complex characteristics of land development trends and city sprawl, this study focuses on the scope of land carrying capacity with a holistic view.

In future work, many scope-related city development and city sprawl issues have to be examined, such as limited growth, ecosystem and carrying capacity, healthy eco-city and eco-community, greenhouse effect, green building, land use management, transportation, and urban design. Accordingly, there are a lot of issues this paper addresses that need to be looked at in small areas or corridors to better understand the effects at the microscopic level, and bring forth the key strategies for implementation in quantitative SD models. All of these need to be considered continually and equally.
